# Verbal Memory Performance and Reduced Cortical Thickness of Brain Regions Along the Uncinate Fasciculus in Young Adult Cannabis Users

**DOI:** 10.1089/can.2017.0030

**Published:** 2018-03-01

**Authors:** Nina Levar, Alan N. Francis, Matthew J. Smith, Wilson C. Ho, Jodi M. Gilman

**Affiliations:** ^1^ Center for Addiction Medicine, Massachusetts General Hospital, Boston, Massachusetts.; ^2^ Department of Psychiatry, Harvard Medical School, Boston, Massachusetts.; ^3^ McLean Imaging Center, McLean Hospital, Belmont, Massachusetts.; ^4^ School of Social Work, University of Michigan, Ann Arbor, Michigan.

**Keywords:** addiction, cannabis, cortical thickness, diffusion tensor imaging, fractional anisotropy, mean diffusivity, verbal memory

## Abstract

**Introduction:** Memory impairment is one of the most commonly reported effects of cannabis use, especially among those who initiate use earlier, perhaps due to the effects of delta-9- tetrahydrocannabinol on cannabinoid (CB1) receptors in the brain. Studies have increasingly investigated whether cannabis use is associated with impairments in verbal memory, and with alterations in brain structures underlying verbal memory. The uncinate fasciculus (UF), a long-range white matter tract, connects regions with densely localized CB1 receptors that are important in verbal memory. This study investigated the impact of cannabis use on UF structures and its association with memory performance in young adult cannabis users (CU) and non-using controls (CON).

**Materials and Methods:** Nineteen CU and 22 CON completed a verbal memory task and a neuroimaging protocol, in which diffusion tensor imaging and structural scans were collected. We compared memory performance, diffusion and tractography measures of the UF, and cortical thickness of regions connected by the UF, between CU and CON. In regions showing a significant group effect, we also examined associations between verbal memory performance, cortical thickness, and age of onset of cannabis use.

**Results:** Compared to non-users, CU had worse memory performance, decreased fiber bundle length in the UF, and decreased cortical thickness of brain regions along the UF such as the entorhinal cortex and fusiform gyrus. Verbal memory performance was significantly associated with age of onset of cannabis use, indicating that those who initiated cannabis use at an earlier age performed worse. Cortical thickness of the entorhinal cortex was significantly correlated with age of first use and memory performance.

**Conclusion:** This study provides evidence that cannabis use, especially when initiated at a young age, may be associated with worse verbal memory and altered neural development along the UF. Reductions in cortical thickness in regions implicated in memory processes may underlie weaknesses in verbal memory performance.

## Introduction

Cannabis is the most commonly used illicit drug among young adults in the United States with 19.8% of 18–25 year olds reporting use in the past month.^1^ Cannabis use has been associated with deficits in several aspects of memory,^2^ particularly with impairments in verbal memory (i.e., memory for words and verbal, rather than spatial, items).^3–9^ Moreover, cannabis use has been related to changes in brain structure, especially among those with an earlier age of cannabis initiation (for reviews, see Refs.^10–13^).

**FIG. 1.**
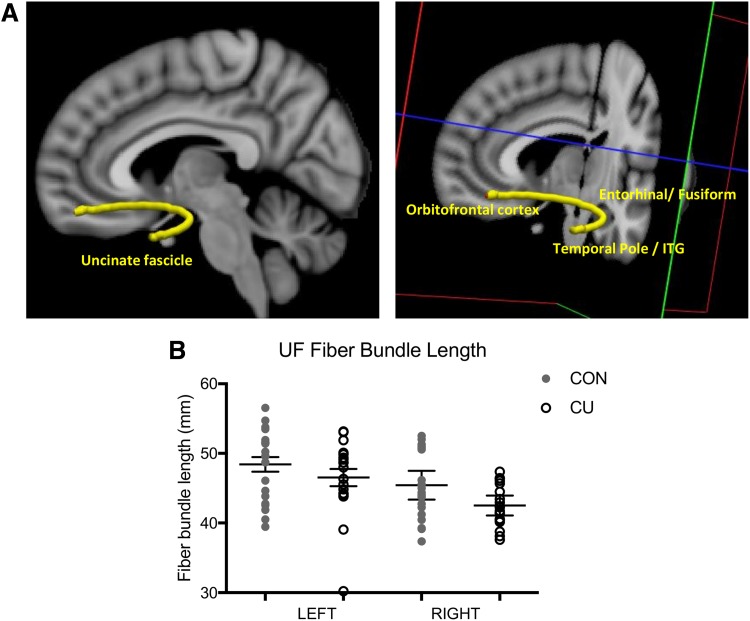
**(A)** Visualization of the bilateral uncinate fasciculus connecting the orbitofrontal poles, temporal poles, entorhinal cortex, fusiform gyri, and inferior temporal gyri, among all participants, using TRACULA. **(B)** Scatterplot of fiber bundle length for the left and right hemisphere for CU and CON. There was a significant reduction in fiber bundle length in the UF in CU compared to controls. CU, cannabis users; UF, uncinate fasciculus.

Delta-9-tetrahydrocannabinol (THC), the main psychoactive compound in cannabis, binds to endogenous cannabinoid (CB1) receptors located in brain regions known to support verbal memory, including the fusiform gyrus, entorhinal cortex, and temporal pole,^14,15^ as well as in other connecting regions that are involved in manipulating and integrating information, such as the inferior temporal lobe and the orbitofrontal cortex (OFC).^16,17^ These structures are connected by the uncinate fasciculus (UF), a crescent shaped, monosynaptic, bidirectional long-range fiber bundle^18,19^ (see Fig. 1A for illustration). The UF and its interconnected structures support encoding and retrieval aspects of verbal memory,^20^ which may be impaired among cannabis users (CU).^21–24^ To our knowledge, studies have not examined potential differences between CU and non-using controls in white matter integrity of UF, cortical thickness of the regions it connects, and their relationship to memory performance.

Generally, cannabis-related alterations in brain structure are greater in individuals with an earlier age of cannabis initiation.^25,26^ Adolescent cannabis exposure has also been associated with compromised white matter integrity,^27–31^ including microstructural abnormalities in the splenium of the corpus callosum and the fornix.^32,33^ Memory impairment is also greater among those with an earlier age of onset of use. Heavy CU show a variety of memory deficits, including poorer immediate and delayed recall,^21–24,34^ and the degree of memory impairments has been associated with duration, quantity, and age of onset of cannabis use.^24^ Our laboratory has extended these findings, reporting that early adolescent onset of cannabis use (e.g., use before the age of 16) is associated with greater verbal memory impairments due to weaknesses in encoding.^35^

Studies have separately shown that cannabis use may reduce cortical thickness^36^ or impair memory performance,^22–24,34,37^ but few have evaluated the relationship between cannabis-related changes in brain structure and memory performance. Generally, there is poor evidence for robust associations between measures of brain morphology and neurocognitive performance (see Ref. 38 for review), although a handful of studies have demonstrated this link (e.g., see Refs. 39 and 40). In this study, we investigated memory performance and structural measures of brain regions implicated in memory in young adult CU, and associated these factors with the age of onset of cannabis use. Specifically, we used diffusion tensor imaging (DTI) imaging, tractography, and structural analyses to examine directional coherence and fiber bundle length of the UF, as well as cortical thickness of regions connected by the UF. We had three main hypotheses: (1) verbal memory performance would be significantly worse in CU compared to CON, (2) directional coherence and fiber bundle length of the UF, as well as cortical thickness of the regions connected by the UF (e.g., medial and lateral parts of OFC, the fusiform gyri, the entorhinal cortex, and the inferior temporal pole), would be different in CU compared to CON, and (3) worse memory performance would be more pronounced in the CU group among those with an earlier age of cannabis initiation. As a *post-hoc*, exploratory analyses, we also investigated correlations among structural measures, verbal memory performance, and age of cannabis initiation.

## Materials and Methods

### Participants

Forty-one young adults between 18 and 25 years (M=21.12; SD=2.26) participated in this study. Nineteen (8 male and 11 female) were recreational CU and 22 (10 male, 12 female) were non-using controls (CON) (Table 1). Participants were medically healthy, and did not meet *Diagnostic and Statistical Manual of Mental Disorders, 4th Edition* (DSM-IV) criteria for any current or lifetime Axis I disorders according to the Structural Clinical Interview of the DSM-IV Disorders (SCID-4) (except for cannabis abuse or dependence in the CU group). CU and CON were matched on age, gender, handedness, race, and years of education. All participants were right handed. Participants completed the AUDIT (Alcohol Use Disorder Identification Test)^41^ to assess alcohol drinking behaviors. No participant met criteria for hazardous drinking behavior, defined by scores above 16, and none were regular cigarette smokers. CU participants used cannabis at least once a week (M=5.87 joints/week). Past experimental use of other illegal drugs did not lead to exclusion; however, participants were excluded from the study if they had ever met abuse criteria for any other drug than cannabis. Three CU reported illicit drug use: one reported cocaine use, one reported ecstasy use, and one reported use of cocaine, mushrooms, lysergic acid diethylamide, and N,N-dimethyltryptamine. No participant reported any drug use of more than five times in their lifetime. CON were included in this study if they had not used cannabis in the past 6 months, and had used cannabis on less than five occasions in their lifetime. All participants gave written informed consent and were compensated for their time. Experiments were approved by the Partners Human Research Committee Institutional Review Board at Massachusetts General Hospital.

**Table 1.** **Participant Demographics**

	Cannabis users (*n*=19)	Controls (*n*=22)
Gender (m/f)	8/11	10/12
Age (years)	20.58 (2.52)	21.59 (1.94)
Years of education	14.16 (1.80)	15.05 (1.56)
No. of alcoholic drinks/week^*^	3.03 (2.14)	1.90 (2.33)
Cannabis use (days/week)	2.70 (1.48)	n/a
No. of joints/week	5.87 (7.36)	n/a
No. of days from last cannabis use	2.79 (3.10)	n/a
Age of first cannabis use (years)	16.21 (1.69)	n/a
Duration of use (years)	4.37 (1.67)	n/a

All values are means and standard deviations.

^*^ *p*<0.05.

#### Procedures

Participants completed two study visits: a screening visit, in which they completed cognitive testing, and a second study visit in which they underwent a neuroimaging session. All CU were asked to refrain from using substances on study days, but were permitted to use cannabis up until the night before the study (to reduce possible withdrawal effects). We performed a urine drug screen to test for cannabis, amphetamines, cocaine, barbiturates, methamphetamines, benzodiazepines, codeine, morphine, and ethanol. Since THC-COOH, the main secondary metabolite of tetrahydrocannabinol, can still be detected in urine up to several weeks after last use, we used a four-item cannabis intoxication scale^42^ to rule out acute intoxication. Acute intoxication was determined based on the following criteria: increased resting heart rate (100 beats per minute), congestions of the conjunctival blood vessels (red eyes), slowed speech responses, and giddiness. No participants met criteria for acute intoxication. CU participants were asked to complete a timeline followback questionnaire^43^ to retroactively establish the number of days that they used cannabis, as well as the number of joints they smoked per occasion, and the number of smoking events per day, for the past 90 days. Both CU and CON also completed a timeline followback questionnaire assessing alcohol use and drinking behavior over the past 90 days.

#### Memory testing

All participants completed the California Verbal Learning Test Second Edition (CVLT-II; Ref.^44^), which involves verbal presentation of a 16-word list consisting of 4 nonadjacent words from four different semantic categories (i.e., vegetables, modes of travel, animals, and furniture). The list is presented five consecutive times, and participants recalled the words after each learning trial. After a 20-min delay, participants recalled as many words as they could remember. Primary outcome variables included the following: short delayed free recall (SDFR; total words freely recalled immediately; range: 0–16), long delayed free recall (LDFR; total words freely recalled after a 20-min delay; range: 0–16), short delayed cued recall (SDCR; total words recalled immediately when presented with cued categories; range: 0–16), and long delayed cued recall (LDCR; total words recalled after a 20-min delay with cued categories; range: 0–16). We also computed scores for Trial 1 recall, total learning, learning slope, and semantic and serial clustering. Raw scores were converted to standardized scores for analyses.

#### Image acquisition and analyses

Neuroimaging data were acquired using a 3T Siemens (Erlangen/Germany) Trio scanner with a 32-channel head coil at the Martinos Center for Biomedical Imaging. Whole-brain T1-weighted 1 mm isotropic structural scans were collected using a 3D multi-echo MPRAGE sequence (176 sagittal slices, 256 mm FoV, repetition time (TR)=2530 msec, TI=1200 msec, 2×GRAPPA acceleration, echo time (TE)=1.64/3.5/5.26/7.22 msec, BW 651 Hz/px, T_acq_=6.03 min).^45^ Diffusion-weighted images were acquired using single spin-echo echo-planar imaging (EPI) with 10 nondiffusion weighted (b=0 s/mm^2^) images and 2 non-zero b-values (900, 2000 s/mm^2^), each with 60 directions; TR/TE=2400 msec/66 msec, 2.0 mm^3^ isotropic resolution, 256 mm FOV, total scan time=3:24 min:sec. All raw and processed data were visually inspected and determined to be of good-to-excellent quality. Images were aligned and registered to the MNI152 2 mm^3^ standard space template (Montreal Neurological Institute, Montreal, QB, Canada).

#### DTI and quantitative diffusion tractography

Diffusion-weighted images were acquired and a total of 64 echoes was collected, with echo spacing of 0.78 msec and a readout bandwidth of 1490 Hz/px, resulting in a total echo train length of 84.42 msec. DTI data were analyzed using the TRACULA package, integrated in the Freesurfer Software version 5.3, which reconstructs white matter pathways using global probabilistic tractography. First, we performed an automated reconstruction followed by automated labeling of cortical and subcortical regions on the anatomical T1-weighted images, which FMRIB Software Library (FSL) performs based on probabilistic information from a manually labeled training set. Next, in compliance with standard TRACULA preprocessing procedures, we applied FSL's eddy-current correction algorithm to correct for motion and eddy-current effects. For quality assessment, four measures of head movement were calculated from the diffusion weighted imaging (DWI) and the output of the prior eddy-current correction procedures (average translation, average rotation, percent “bad” slices, and percent dropout score). Any participant with >1.5 mm of moment (translation or rotation) or with >2% of bad slices or dropout was excluded. There was no significant difference in motion between groups (all *p'*s>0.10), and no participants were excluded due to excessive motion. We then performed an affine registration of structural T1 images and diffusion tensors of each participant using bbregister for intra-subject registration. Before diffusion-to-template transformations, T1 images of all participants were registered to the MNI152 2 mm^3^ standard space template. Subsequently, tensors were estimated and then mapped from diffusion space to MNI space. TRACULA then applied the ball-and-stick model of diffusion to the data and provided the reconstructed pathways. In a last step, tracts were estimated and fitted by combining the output of the ball-and-stick model and the atlas data obtained from manually labeled tracts. The final output for the bilateral UF was assessed for mean diffusion (MD), radial diffusivity (RD), axial diffusivity, (AD), and fractional anisotropy (FA), defined as the fraction of the magnitude of the tensor that is due to anisotropic water diffusion. For each tract, we computed mean values from all voxel across the tract, and determined fiber bundle length. An illustration of the UF in all participants was generated using TRACULA.

#### Cortical thickness

T1-weighted images were analyzed using Freesurfer (Martinos Center for Biomedical Imaging, Massachusetts General Hospital, Boston; http://surfer.nmr.mgh.harvard.edu). Images were aligned and registered to the MNI152 2 mm^3^ standard space template (with 0.7 mm resolution) and corrected for spatial distortion and smoothed using an full width at half maximum (FWHM) of 5 mm. Whole brain segmentation and cortical parcellation were applied to the corrected T1-weighted images.^46,47^ We visually inspected processed images and segmentations, and any errors in processing were corrected using manual methods. The edited scans were then reprocessed and cortical thickness data were extracted from the corrected images. We examined cortical thickness of six regions along the UF: medial OFC, lateral OFC, fusiform gyrus, entorhinal cortex, temporal pole, and inferior temporal pole. Measures of additional brain volumes (e.g., amygdala and hippocampus) were also extracted, but were not a primary focus of this report.

#### Statistical methods

Group differences in background characteristics were assessed using Student's *t*-tests in SPSS version 19. All statistical tests were two tailed. For memory performance data, we ran a multivariate ANCOVA, including all memory variables as dependent variables, group as an independent variable, and alcohol as a covariate. For all structural data (diffusion and cortical thickness), we ran a series of repeated-measures (RM) ANCOVAs, using hemisphere as a repeated measure and alcohol (drinks per week) and gender as covariates. In addition, we used intracranial volume as a covariate when analyzing tract length, as tract length likely scales with head size. These RM-ANCOVAs assessed for a group effect of CU versus CON, as well as a group×hemisphere effect to determine if group differences were affected by hemisphere. We used false discovery rate (*q*=0.05) to control for multiple comparisons^48^ among the six bilateral structures assessed (medial OFC, lateral OFC, fusiform gyrus, entorhinal cortex, temporal pole, and inferior temporal pole), and for three diffusion measures assessed bilaterally (FA, MD, and fiber bundle length). We also calculated effect sizes of each region on each side separately, using Cohen's *d*. Significant ANCOVAs were followed by *post-hoc* Tukey's honest significant difference (HSD) tests to isolate effects. In any structural measurement (DTI or cortical thickness) that showed a significant difference between CON and CU, we performed Pearson correlations between the structural measure and memory performance.^49^ Data were checked for normality and analyzed for outliers using the robust regression and outlier removal (ROUT) method.^49^

## Results

### Participants

CU and controls did not significantly differ in age, gender, or years of education (Table 1). CU used cannabis on an average of 2.70 (SD=1.48) days per week and 1.66 (SD=0.94) times per day, and smoked 5.87 (SD=7.36) joints per week. The average duration of cannabis use in CU was 4.37 (SD=1.67) years. Recency of use was 2.79 (SD=3.10) days on average. There was a significant group difference in alcohol consumption per week (*p*=0.04; CU: M=3.03, SD=2.14; CON: M=1.90, SD=2.33). Therefore, all analyses controlled for alcohol use.

### Memory performance

**FIG. 2.**
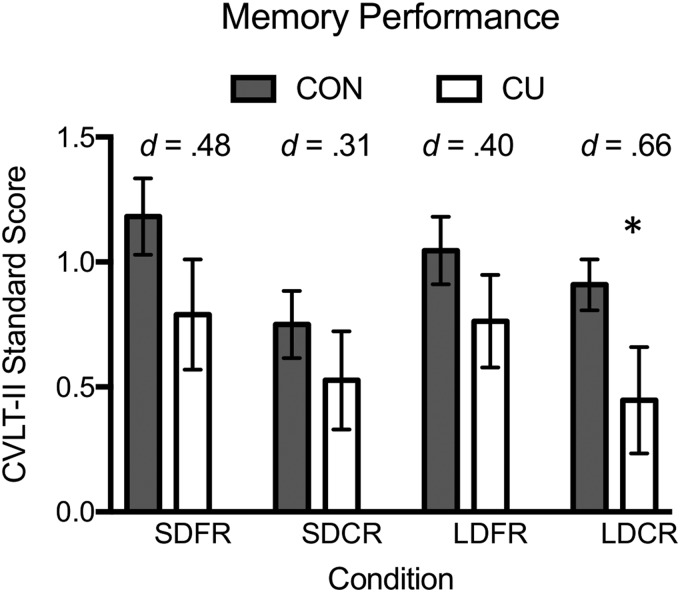
California verbal learning task performance. Bar plots show standardized CVLT scores of CU and CON for each of four conditions: SDFR, SDCR, LDFR, and LDCR. There was a significant effect of group across conditions, with CU performing significantly worse than CON (*F*4.36)=2.7, *p*=0.04). A *post-hoc* analysis showed that only LDCR was significantly different between groups. Effect sizes (Cohen's *d*) are shown for each condition. *Significant differences between groups using a multivariate ANCOVA (*p*<0.05). CVLT, California Verbal Learning Test; SDFR, short-delay free recall; SDCR, short-delay cued recall; LDFR, long-delay free recall; LDCR, long-delay cued recall.

Across all recall measures of the CVLT-II (SDFR, SDCR, LDRF, and LDCR), there was a significant effect of group (*F*^4,36^=2.7, *p*=0.038, *ηp^2^*=0.23), indicating that CU performed significantly worse than CON across these conditions (Fig. 2). *Post-hoc* tests indicated a significant difference in LDCR between CON and CU (*t*=2.05, *p*=0.04, Cohen's *d*=0.66). For the other memory measures, CU generally performed worse than CON, indicated by small to medium effect sizes, but between-group differences were nonsignificant (SDFR: *t*=1.49, *p*=0.14, Cohen's *d*=0.47; SDCR: *t*=0.96, *p*=0.34, Cohen's *d*=0.31; LDFR: *t*=1.25, *p*=0.21, Cohen's *d*=0.40). Additional measures of memory performance were not significantly different between groups (Supplementary Table S1).

### Quantitative tractography

Using RM ANCOVAs, we found that CON and CU did not show any significant differences in FA, mean diffusivity, RD, or AD of the UF through either a main effect of group or a group×hemisphere interaction (all *p*>0.10, see Supplementary Table S2). For an illustration of the UF in all participants, see Figure 1A. For fiber bundle length, one CU participant was determined to be an outlier and was therefore removed from analyses. After controlling for intracranial volume, we observed a main effect of group on UF bundle length (*F*=4.27, *p*=0.04), which revealed that length was significantly reduced in CU compared to CON (Fig. 1B). There was no group×hemisphere interaction (*F*=0.35, *p*=0.55).

### Cortical thickness

Using RM ANCOVAs, we investigated group differences in the six regions connected to the UF. In the frontal extension, we found a significant main effect of group on the lateral OFC (*F*=5.30, *p*=0.027), but not the medial OFC (*F*=1.77, *p*=0.19). In the middle segment, we found significant main effects of group on the fusiform gyrus (*F*=6.97, *p*=0.01) and entorhinal cortex (*F*=7.27, *p*=0.01). In the temporal segment, we found a significant main effect of group on the temporal pole (*F*=4.13, *p*=0.049), but not the inferior temporal cortex (*F*=2.21, *p*=0.15). After adjusting for false discovery rate (*q*=0.05), only the main effects on the middle segments (i.e., fusiform gyrus and the entorhinal cortex) remained significant. None of these regions showed significant group×hemisphere interactions (all *p*>0.10). See Table 2 for mean values and effect sizes. We further investigated group differences in regions previously implicated in cannabis use, such as the hippocampus, thalamus, and amygdala. Only the right amygdala was significantly different between CON and CU (*F*=4.39, *p*=0.04) (Supplementary Table S3).

**Table 2.** **Fiber Bundle Length and Cortical Thickness of Regions Along the Uncinate Fasciculus**

		Controls (22)		CU (19)		
Region	Hemi	Mean	SD	Mean	SD	Cohen's *d*
Fiber bundle length^a^	Left	48.4	4.96	46.5	5.4	0.38
	Right	45.40	4.65	42.50	2.94	0.75
Medial OFC	Left	2.55	0.15	2.50	0.14	0.35
	Right	2.50	0.15	2.41	0.13	0.65
Lateral OFC^a^	Left	2.83	0.14	2.74	0.12	0.70
	Right	2.72	0.15	2.6	0.15	0.82
Fusiform gyrus^b^	Left	2.83	0.14	2.74	0.13	0.68
	Right	2.90	0.11	2.81	0.11	0.84
Entorhinal gyrus^b^	Left	3.60	0.32	3.44	0.43	0.44
	Right	3.90	0.21	3.65	0.32	0.96
Temporal pole^a^	Left	3.90	0.32	3.73	0.21	0.63
	Right	4.00	0.28	3.94	0.25	0.23
Inferior temporal gyrus	Left	3.02	0.14	2.90	0.19	0.75
	Right	3.01	0.14	2.93	0.19	0.50

Fiber bundle length, mean values, standard deviations, and effect sizes of cortical thickness of regions along the UF.

^a^ Significant differences between groups using a repeated measures ANCOVA.

^b^ Significant differences between groups after FDR correction.

CU, cannabis users; FDR, false discovery rate; UF, uncinate fasciculus; OFC, orbitofrontal cortex.

### Associations between cannabis use, brain structure, and memory performance

**FIG. 3.**
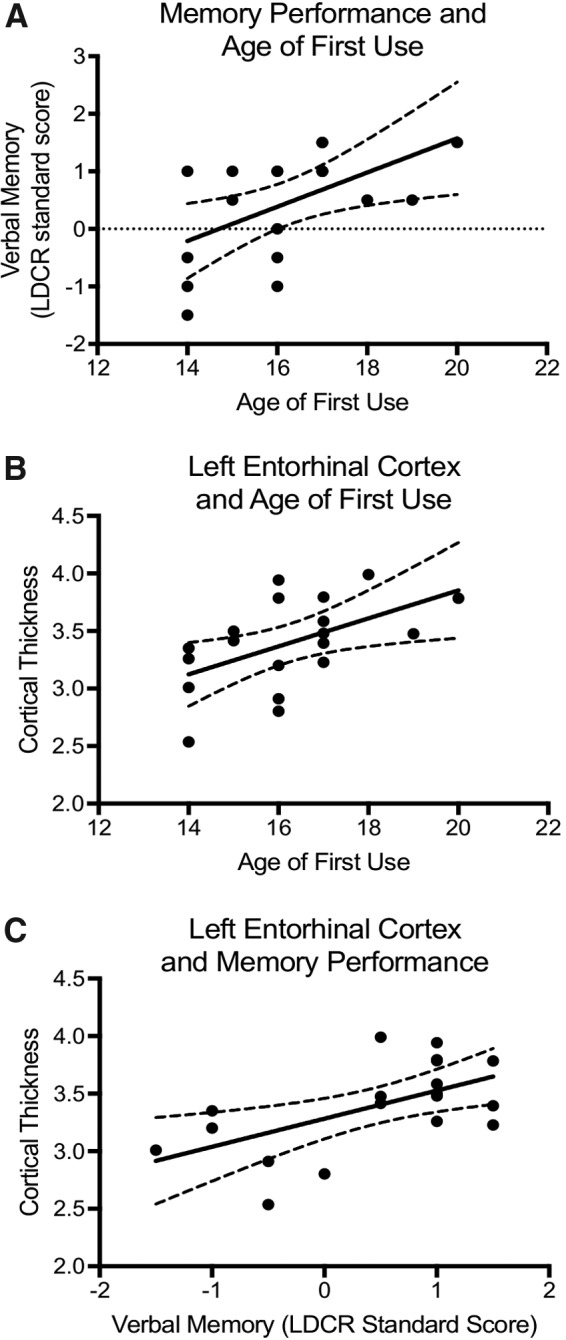
**(A)** Age of first cannabis use was positively correlated with average standardized score on the CVLT-II (*r*=0.59, *p*=0.008). **(B)** Cortical thickness in the left entorhinal cortex was significantly correlated with age of onset of cannabis use (*r*=0.53, *p*=0.02). **(C)** Cortical thickness in the left entorhinal cortex was significantly correlated with average memory performance (*r*=0.58, *p*=0.009). Each point represents one participant, and the 95% confidence interval is depicted.

Age of first use of cannabis was significantly associated with memory performance on LDCR (*r^2^*=0.29, *p*=0.016), indicating that individuals who had earlier use performed worse than those with later onset of use (Fig. 3A). Based on our previous analyses, we restricted correlations to those structures that showed significant differences between groups: UF fiber bundle length, cortical thickness of the entorhinal cortex, and cortical thickness of the fusiform gyrus. Cortical thickness in the left entorhinal cortex was significantly correlated with age of first use (*r^2^*=0.27, *p*=0.02) (Fig. 3B) and LDCR scores (*r^2^*=0.34, *p*=0.009) (Fig. 3C). UF fiber bundle length and cortical thickness of the fusiform gyrus were not associated with age of first use or with memory performance.

## Discussion

Despite emerging evidence that cannabis use appears to disrupt verbal memory and is associated with alterations of brain structure, few studies have investigated whether cannabis use is associated with altered white matter tracts and cortical thickness in structures that support verbal memory. Thus, this study investigated (1) verbal memory performance in CU and controls, (2) white matter integrity of the UF, and cortical thickness of the regions connected to the UF that support verbal memory (e.g., the OFC, entorhinal cortex, fusiform cortex, and temporal poles), (3) how age of onset of cannabis use affected verbal memory performance, and (4) associations between verbal memory performance, morphometry, and age of initiation of cannabis use. Three main findings emerged from this study. First, memory performance on delayed recall (LDCR) was worse in CU than the CON group. Second, UF fiber bundle length, as well as cortical thickness of several prefrontal and temporal regions along the UF, was reduced in CU. Third, age of onset of cannabis use correlated with memory performance decrements. In *post-hoc* analyses, we also found that decreased cortical thickness was associated with age of onset of cannabis use and verbal memory performance.

Emerging research has shown that cannabis use, particularly during adolescence, is associated with verbal memory deficits.^50^ This period of increased vulnerability of verbal memory processes to cannabis exposure may be due to the normal process of rapid development of the endocannabinoid system that takes place during adolescence.^51,52^ In this study, we observed that the CU group performed worse on LDCR compared to CON, and a younger age of onset of cannabis use was correlated with poorer performance on LDCR. This result is consistent with an emerging literature on the effects of adolescent cannabis use^2,53^ and our prior work demonstrating that delayed recall is compromised in young adults with early-onset cannabis use (<16 years), compared to young adults with late cannabis use onset (>16 years) and control subjects.^35^ It is important to note that our previous study demonstrated that weaknesses in delayed recall were fully mediated by weaknesses in encoding (e.g., Trial 1 learning) among early-onset CU. We did not detect this effect in this study, likely because of the smaller sample size and the mixed age of first use among our participants.

Verbal memory is supported by a wide range of brain regions,^20^ including the prefrontal cortex and regions within the temporal lobe. These regions are rich in cannabinoid CB1 receptors,^54^ which may render them vulnerable to cannabis-related alterations. Verbal memory is also supported by white matter tracts such as the UF, which serves a broad array of brain functions, including memory (value-based updating of stored representations), and language related to verbal memory (retrieval of names; aspects of semantic memory retrieval).^18^ In this study, we observed that the CU group had reduced measures of FA compared to CON that were characterized by a medium effect size. However, these differences did not attain statistical significance. A previous study found reductions in FA and MD in the bilateral UF of regular CU,^55^ while other studies have found significant reductions in the FA and MD in numerous other white matter tracts, including the arcuate fasciculus, and superior longitudinal fasciculus.^28,37,55^ However, these cohorts generally had earlier ages of cannabis use onset or longer duration of cannabis use than the participants in this study. The observed group differences in these studies could indicate that alterations in FA and MD values may become more pronounced with prolonged consumption and earlier onset of use, or in older, more chronic CU. Alternatively, the lack of significance in this study could be explained by limited statistical power due to the size of our study sample. We found that UF fiber bundle length was shorter in CU compared to CON, which may indicate that fiber bundle length may be related to lighter patterns of cannabis use. We did not detect associations between white matter measurements and age of onset of use. It is important to note that white matter volume does not reach its peak until adulthood, between the mid-30s and 40s^56–58^; so it is possible that cannabis use could affect UF development throughout life.

We found significantly reduced cortical thickness in CU compared to CON in the entorhinal cortex and fusiform gyrus, which are regions connected by the UF^18,19^ and known to support verbal memory.^20^ To our knowledge, studies have not yet investigated cannabis use and cortical thickness in these temporal regions. The entorhinal and fusiform cortices are dense with CB1 receptors, and therefore, this finding is consistent with studies reporting reduced cortical thickness in other regions dense in CB1 receptors, such as the prefrontal cortex.^25^ Moreover, we found that the entorhinal cortex was associated with memory performance and age of onset of cannabis use. Although the entorhinal cortex is generally not as well studied as regions such as the hippocampus or prefrontal cortex for memory function, there is literature suggesting role for the entorhinal cortex in memory. For example, in a study of healthy adults, longitudinal changes in entorhinal cortex volumes were shown to predict verbal memory performance.^59^ However, it is important to note that the association reported in this study was found in a small sample size, and should therefore be replicated in larger samples.

In conclusion, this study reports significant group differences between CU and CON in verbal memory performance and brain-based structural measures of the UF, and cortical thickness in several of its connecting regions. Results of this study should be interpreted cautiously. First, as with all cross-sectional studies, effects described could have been pre-existing, a result of cannabis use, or a combination of both. Second, our participants used alcohol, which is a common confounding factor in magnetic resonance imaging (MRI) studies of cannabis exposure. Thus, we controlled for alcohol consumption in our statistical analyses; however, future studies could attempt to match participants for alcohol use measures. To this end, we caution that correlations between LCDR performance and cortical thickness may not be specific to cannabis use. Third, the small sample size limits our ability to conduct detailed subanalyses (e.g., analyses based on early and late cannabis use onset, gender, or frequency/duration of use), and we likely had limited power to detect memory performance effects in encoding. Fourth, there may have been methodological limitations to this study (e.g., the analysis could not control for all brain inhomogeneity artifacts). Last, as cannabis remains in the system for up to a month, it is possible that the reported effects on memory are due to recent cannabis use. Thus, the observed group differences could be, in part, explained by residual effects of circulating cannabis metabolites in the CU group. Future studies can examine how the above factors relate to group differences between CU and CON. Furthermore, future research can also extend investigations to other white matter tracts to more thoroughly understanding the impact of cannabis exposure on the human brain and its potential effects on cognitive processing.

## Supplementary Material

Supplemental data

Supplemental data

Supplemental data

## Abbreviations Used

AD: axial diffusivity
CB1: cannabinoid
CU: cannabis users
CVLT: California Verbal Learning Test
DTI: diffusion tensor imaging
FA: fractional anisotropy
LDCR: long delayed cued recall
LDFR: long delayed free recall
MD: mean diffusion
OFC: orbitofrontal cortex
RD: radial diffusivity
RM: repeated measures
SDCR: short delayed cued recall
SDFR: short delayed free recall
THC: delta-9-tetrahydrocannabinol
UF: uncinate fasciculus
