# Supplementary material for: Verbal Memory Performance and Reduced Cortical Thickness of Brain Regions Along the Uncinate Fasciculus in Young Adult Cannabis Users
Source: Cannabis Cannabinoid Res. 2018 Mar 1;3(1):56–65. doi: 10.1089/can.2017.0030 (PMC5870060; doi:10.1089/can.2017.0030)
Supplement: Supplemental data [file Supp_Table1.pdf]

Supplementary Data

Supplementary Table S1. Neurocognition

	CON		CU			
**Neurocognition**	**Mean**	**SD**	**Mean**	**SD**	***t* Score**	***p***
Trial 1 recall						
Raw	9.57	2.52	9.21	3.46	0.38	0.71
Z Score	1.24	1.40	0.92	1.87	0.61	0.55
Total learning						
Raw	66.71	7.34	63.05	10.98	1.25	0.22
T score	65.43	12.10	62.53	13.90	0.71	0.49
Learning slope						
Raw	1.22	0.61	1.23	0.69	−0.04	0.97
T Score	−0.40	1.03	−0.37	1.29	−0.10	0.92
Semantic clustering						
Raw	3.04	3.10	2.29	3.16	0.76	0.46
Z score	1.33	1.88	0.95	1.91	0.64	0.52
Serial clustering						
Raw	1.12	2.24	1.59	2.54	−0.62	0.53
Z score	0.36	1.97	0.53	1.86	−0.28	0.78
Short-delay free recall						
Raw	14.48	1.72	13.32	2.96	1.53	0.13
Z Score	1.12	0.71	0.71	1.08	1.43	0.16
Short-delay cued recall						
Raw	14.52	1.72	13.53	2.72	1.40	0.17
Z Score	0.71	0.62	0.39	1.01	1.22	0.23
Long-delay free recall						
Raw	14.81	1.57	13.79	2.86	1.42	0.16
Z score	1.00	0.63	0.63	1.03	1.38	0.17
Long-delay cued recall						
Raw	15.10	1.26	13.74	2.77	2.03	0.04*
Z score	0.86	0.48	0.37	1.07	1.90	0.06
Percent retention	98.11	4.22	93.70	10.46	1.78	0.08

Mean values, standard deviations, and statistical significance of CVLT performance in cannabis users and controls.

*Significant differences between groups using a multivariate ANCOVA ($p < 0.05$).
