# Supplementary material for: Verbal Memory Performance and Reduced Cortical Thickness of Brain Regions Along the Uncinate Fasciculus in Young Adult Cannabis Users
Source: Cannabis Cannabinoid Res. 2018 Mar 1;3(1):56–65. doi: 10.1089/can.2017.0030 (PMC5870060; doi:10.1089/can.2017.0030)
Supplement: Supplemental data [file Supp_Table2.pdf]

Supplementary Table S2. Quantitative Diffusion Imaging Measures of the Uncinate Fasciculus

Measure	Hemi	CON (22)		CU (19)		Cohen's *d*
		Mean	SD	Mean	SD	
FA	Left	0.40	0.04	0.38	0.05	0.46
	Right	0.38	0.03	0.37	0.02	0.40
MD	Left	0.0008	0.001	0.0008	0.001	0.00
	Right	0.0008	0.001	0.0009	0.001	0.10
RD	Left	0.0067	0.00003	0.0067	0.00003	0.00
	Right	0.0008	0.001	0.0009	0.001	0.00
AD	Left	0.0012	0.0001	0.0012	0.0001	0.00
	Right	0.0012	0.00005	0.0012	0.00003	0.00

For each tract, mean values were computed from all voxel across the tract. CON and CU did not show any significant differences in FA or MD.

CON, controls; CU, cannabis users; FA, fractional anisotropy; MD, mean diffusivity; RD, radial diffusivity; AD, axial diffusivity.
