# Supplementary material for: Verbal Memory Performance and Reduced Cortical Thickness of Brain Regions Along the Uncinate Fasciculus in Young Adult Cannabis Users
Source: Cannabis Cannabinoid Res. 2018 Mar 1;3(1):56–65. doi: 10.1089/can.2017.0030 (PMC5870060; doi:10.1089/can.2017.0030)
Supplement: Supplemental data [file Supp_Table3.pdf]

Supplementary Table S3. Volumes

	Controls		Cannabis users		Variance accounted for by ICV		Variance accounted for by sex		Variance accounted for by group	
	**Mean**	**SD**	**Mean**	**SD**	**F**	***p***	**F**	***p***	**F**	***p***
Estimated ICV	1616024.29	205515.98	1572702.54	137800.56	—	—	7.53	0.01	0.47	0.50
Total gray matter	688758.35	64517.44	669445.72	47956.21	52.54	0.00	0.29	0.59	0.52	0.48
Total white matter	462840.56	63913.98	461836.64	49966.63	48.00	0.00	0.12	0.73	0.72	0.40
Left thalamus	8281.49	981.21	8231.26	694.92	26.62	0.00	0.18	0.67	0.32	0.57
Left caudate	3910.02	642.63	3764.93	422.91	9.55	0.00	0.05	0.82	0.41	0.53
Left putamen	6174.93	714.29	5992.92	530.11	9.04	0.00	0.02	0.89	0.33	0.57
Left hippocampus	4399.36	499.04	4351.02	366.73	5.56	0.02	0.01	0.91	0.01	0.93
Left amygdala	1741.63	250.24	1760.28	144.83	12.48	0.00	0.35	0.56	0.59	0.45
Left accumbens	644.56	147.51	645.96	118.78	4.61	0.04	1.90	0.18	0.26	0.61
Right thalamus	7469.87	736.75	7280.08	588.55	20.74	0.00	0.02	0.90	0.14	0.71
Right caudate	4072.73	642.22	3988.22	401.76	11.15	0.00	0.08	0.78	0.04	0.84
Right putamen	6044.33	677.56	5878.20	451.83	4.93	0.03	0.44	0.51	0.25	0.62
Right hippocampus	4556.56	509.60	4481.55	338.69	8.25	0.01	0.97	0.33	0.00	0.95
Right amygdala	1717.33	234.59	1785.59	169.40	23.83	0.00	0.21	0.65	4.39	0.04*
Right accumbens	733.60	128.62	718.35	112.01	2.51	0.12	0.04	0.85	0.01	0.92

Cortical thickness of regions previously indicated in memory in cannabis users and controls.
*Significant differences between groups using a repeated-measures ANCOVA ($p < 0.05$).
ICV, intracranial volume.
